# Direct electrodeposition of cationic pillar[6]arene-modified graphene oxide composite films and their host–guest inclusions for enhanced electrochemical performance†

**DOI:** 10.1039/d0ra03138k

**Published:** 2020-06-09

**Authors:** Qunpeng Duan, Lijie Wang, Fei Wang, Hongsong Zhang, Kui Lu

**Affiliations:** School of Materials and Chemical Engineering, Henan University of Engineering Zhengzhou 450006 China qpduan@haue.edu.cn; School of Chemical Engineering and Food Science, Zhengzhou Institute of Technology Zhengzhou 450044 China luckyluke@haue.edu.cn

## Abstract

In the present work, electrochemically reduced graphene oxide-cationic pillar[6]arene (ErGO-CP6) composite films on glassy carbon electrodes (GCEs) were prepared directly from graphene oxide-cationic pillar[6]arene (GO-CP6) dispersions by a facile one-step pulsed electrodeposition technique. The preparation of GO-CP6 and its subsequent electrochemical reduction were confirmed by Fourier transform infrared (FTIR) spectroscopy, UV-vis spectroscopy (UV-vis), thermogravimetric analysis (TGA), X-ray photoelectron spectroscopy (XPS), zeta potential, Raman spectroscopy, and scanning electron microscopy (SEM). SEM result reveals that ErGO-CP6 could form a homogeneous film when GO-CP6 was electrodeposited on the surface of a GCE. Furthermore, Raman and XPS results confirm the removal of oxygen-containing functional groups present on the GO-CP6 surface after electrochemical reduction. Electrochemical results reveal that ErGO-CP6 films could show much higher electrochemical response to theophylline (TP), ascorbic acid (AA), acetaminophen (APAP), and folic acid (FA) than unmodified ErGO films and bare GCE, which is considered to be the synergistic effect of the graphene (excellent electrical properties and large surface area) and CP6 molecules (high inclusion complexation and enrichment capability).

## Abstract 2

Cationic pillar[6]arene functionalized graphene films with enhanced host–guest electrochemical recognition performance were fabricated directly from GO-CP6 dispersions by a one-step pulsed electrodeposition technique.

## Graphical abstract

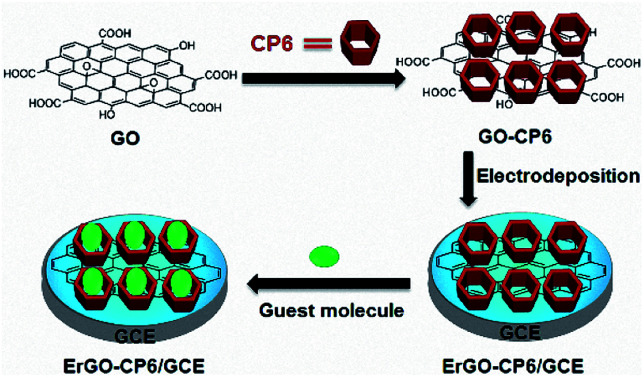

## Introduction

Graphene, a two-dimensional sp^2^-hybridized carbon nanomaterial, has attracted intense scientific interest since its discovery in 2004. It is described as the World's thinnest material, and it presents fascinating mechanical, electronic, thermal, optical, and chemical properties that have made it a promising material for potential use in various fields, such as nanoelectronics,^1^ supercapacitors,^2^ batteries,^3^ sensors,^4^ and nanocomposites.^5^ Reduced graphene oxide (rGO) is the product of reducing graphene oxide (GO) with reducing agents. Although rGO has a relatively lower conductivity than that of the graphene made by the mechanical cleaving method, it is nevertheless a versatile material. In particular, it can be used as a perfect candidate for carbon-based electrode materials to produce electrochemical sensors or biosensors owing to its large active surface area, good electrical conductivity and electrocatalytic activity.^6,7^ However, practical applications of rGO still present a great challenge due to its irreversible agglomeration in aqueous solution, which significantly reduces its effectiveness. Interestingly enough, the introduction of water-soluble macrocyclic hosts as functional molecules can effectively disperse graphene, and further introduce new or enhanced functions through combining their individual characteristics. Therefore, macrocyclic-host-functionalized rGO nanocomposites that simultaneously provide the unique properties of rGO (a large surface area and good conductivity) and the macrocyclic-host (high supramolecular recognition and good enrichment capability) have been extensively exploited as electrocatalysts for improving analyte detection sensitivity.^8–17^ The most commonly used method for the preparation of the macrocyclic-host-functionalized rGO nanocomposite modified electrode is the drop-casting of chemically reduced graphene oxide-macrocyclic host suspension onto the electrode surface. Obviously, such a preparation methodology involves highly toxic chemicals, such as hydrazine hydrate and, moreover, chemical reduction of the graphene oxide-macrocyclic host suspension cannot completely reduce oxygen-containing functional groups, which may result in a decrease in the electrochemical performance.

In more recent times, electrochemical reduction of GO to rGO has attracted considerable attention because it is regarded as a simple, fast and green method in addition graphene film can be obtained by this method on conductive substrates.^18–20^ More importantly, the high negative potential employed in the electrochemically reduced graphene oxide (ErGO) can efficiently reduce the oxygen-rich functional groups present on the GO surface.^21^ Typically, the ErGO has usually been obtained through a two-step procedure comprising the immobilization of GO on the electrode surface by the chemical solution deposition methods, followed by the electrochemical reduction. Up to now, various electrochemical methods including cyclic voltammetry (CV),^22^ potentiostatic method,^23^ and electrophoretic deposition^24^ have been employed. However, the pulsed electrodeposition method,^25^ having some advantages of simplicity, cost efficiency, time saving, and producing high-purity deposits, has rarely been applied in the ErGO field till now.

Pillararenes,^26–34^ as a relatively new class of supramolecular macrocyclic hosts, have attracted continuous attention because of their symmetrical rigid pillar-shaped structures, tunable cavity size, easy functionalization, and unique host–guest recognition abilities. Practically, a series of pillararenes with good water solubility and recognition capability have been applied to fabricate graphene hybrids to improve their water stability and dispersity, as well as to enhance their supramolecular recognition capability in many applications, including sensors, luminescence, electrocatalysis and electronics, and therefore attracted wide research interest.^16,35–37^ Recently, a water-soluble cationic pillar[6]arene (CP6) with twelve –NH_3_^+^ groups on both rims was designed and synthesized by our group.^38^ The CP6 contains twelve hydrophilic ammonium groups on both rims, which can produce electrostatic interaction with the negatively charged groups exist in GO to form GO-CP6 nanocomposites with potential use in materials science.

**Scheme 1:**
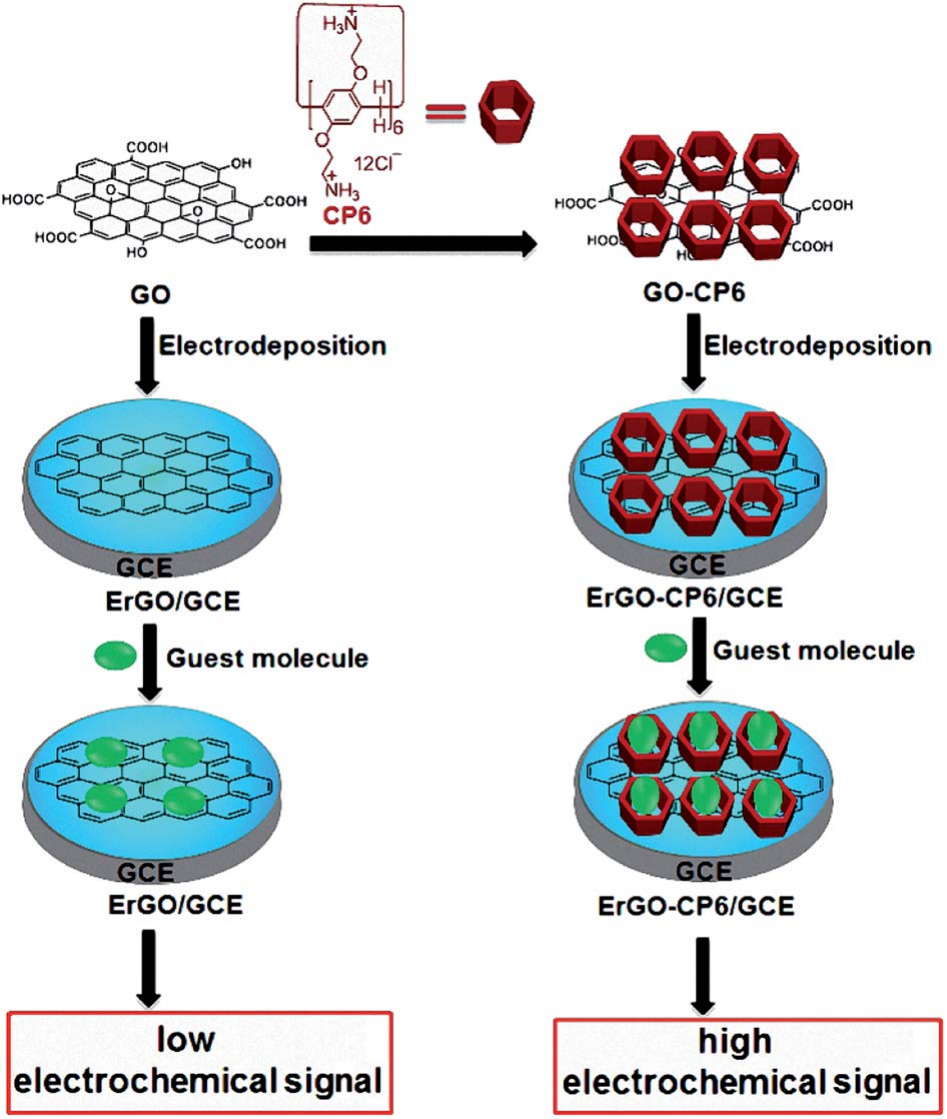
Schematic illustration for the pulsed electrodeposition preparation of ErGO and ErGO-CP6 films on the surface of GCE and sensing the guest molecules by an electrochemical strategy.

In this work, we report for the first time preparation of CP6 functionalized graphene films on glassy carbon electrode (GCE) directly from GO-CP6 dispersions by facile one-step pulsed electrodeposition technique (Scheme 1). The electrodeposited nanocomposite films were characterized by scanning electron microscopy (SEM), Raman spectra, and cyclic voltammetry (CV). The electrochemical performance of the present ErGO-CP6-modified GCE (ErGO-CP6/GCE) was examined by taking theophylline (TP), ascorbic acid (AA), acetaminophen (APAP), and folic acid (FA) as the analytes. The electrochemical behaviors of the four molecules at the ErGO-CP6/GCE displayed much higher electrochemical performance than at those of ErGO/GCE and bare GCE, indicating that the CP6-modified graphene films not only show the outstanding electrical properties of graphene but also exhibit high inclusion complexation and enrichment capability of CP6 through the formation of host–guest inclusion complexes between CP6 and the four molecules.

## Results and discussion

### Characterization of GO-CP6 composites

**Fig. 1:**
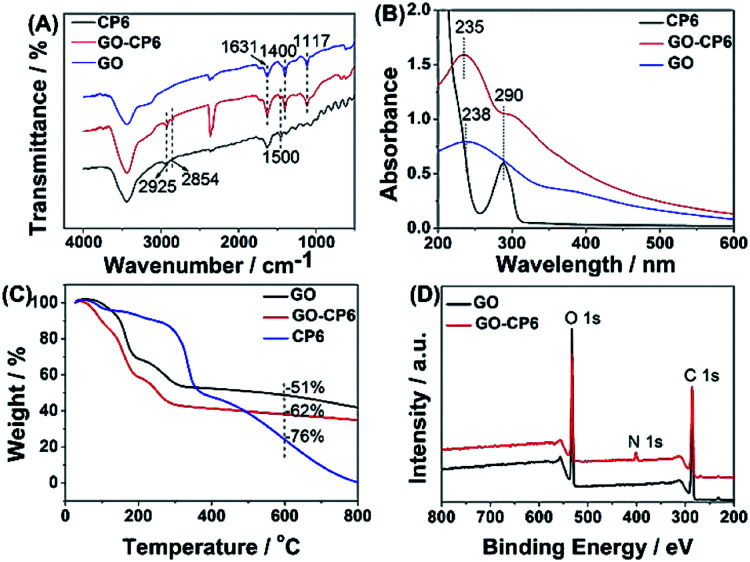
Characterization of materials. FTIR spectra (A), UV-vis absorption spectra (B), TGA curves of CP6, GO-CP6, and GO (C), and XPS survey spectra of GO and GO-CP6 (D).

The FTIR spectra provide clear evidence for the successful functionalization of GO with CP6 (Fig. 1A). As shown in Fig. 1A, the stretching vibrations of –OH (3436 cm^−1^), C=C (1631 cm^−1^), C–OH (1400 cm^−1^), and C–O (1117 cm^−1^) are observed in FTIR spectrum of GO. In the spectrum of GO-CP6, the new bands observed at 2925 and 2854 cm^−1^ correspond to asymmetric and symmetric CH_2_ stretching vibrations, respectively, and the bands centered at 1500 cm^−1^ are observed which are assigned to the typical CP6 absorption features of the phenyl stretching vibrations, indicating that the CP6 molecules have successfully self-assembled on GO and non-covalently formed GO-CP6 composites.^39^ We speculated that the self-assembly mechanism of CP6 on the surface of GO might be ascribed to the cooperative interactions of multiple electrostatic interactions and π–π stacking interactions.^8–10^ The data of UV-vis absorption further confirm the successful preparation of GO-CP6 composites. As shown in Fig. 1B, there is a characteristic absorption peak locating at 290 nm for CP6. The absorption peak of GO is about 238 nm. When CP6 was loaded onto GO, GO-CP6 composites present two main absorption peaks, which are assigned to the absorption peaks of CP6 (∼290 nm) and GO (235 nm). Therefore, the successful chemical modification of CP6 on GO is further confirmed by UV-vis absorption spectra.

TGA measurement was further used to determine the mass fraction of CP6 in GO-CP6 composites. As shown in Fig. 1C, pure CP6 slowly decomposed at approximately 300 °C. The GO has a mass loss (51%) because of the pyrolysis of the labile oxygen-containing functional groups. The loss in mass of the GO-CP6 was about 62 wt% at approximately 600 °C. The mass loss caused by CP6 decomposition was evaluated to be 11 wt% by deducting the mass loss of the GO, suggesting that the mass fraction of CP6 molecules loaded on the surface of GO is 11 wt%. This result is exciting because GO loading plentiful CP6 molecules will provide a good opportunity to expand the inclusion complexation and enrichment ability of CP6.

To further illustrate the formation of GO-CP6, XPS analysis was performed to determine the compositions of GO and GO-CP6. As can be seen from Fig. 1D, a significant N1s peak was observed for the GO-CP6 sample, which comes from the –NH_3_^+^ groups of CP6, but there was no N signal on the GO, further revealing the successful loading of CP6 onto GO.

The average zeta potentials of GO and GO-CP6 are −30.9 and 32.8 mV, respectively, as shown in Fig. S1.† Compared to the zeta potential of GO, the zeta potential of GO-CP6 increases by approximately 63.7 mV, and this is induced by the introduced positive charges of –NH_3_^+^ in the CP6 molecule. The introduced positive charges in GO-CP6 facilitate the stability of the nanocomposite owing to the increased repulsion of positive charges. Furthermore, the zeta potential of GO-CP6 is higher than 30 mV, indicating that the stability and dispersion of GO-CP6 are very high.^40^ Therefore, these results of FTIR, UV-vis, TGA, XPS and zeta potential suggest that CP6 has been successfully grafted on the surface of GO.

### Pulsed electrodeposition of ErGO and ErGO-CP6 films on GCE

**Fig. 2:**
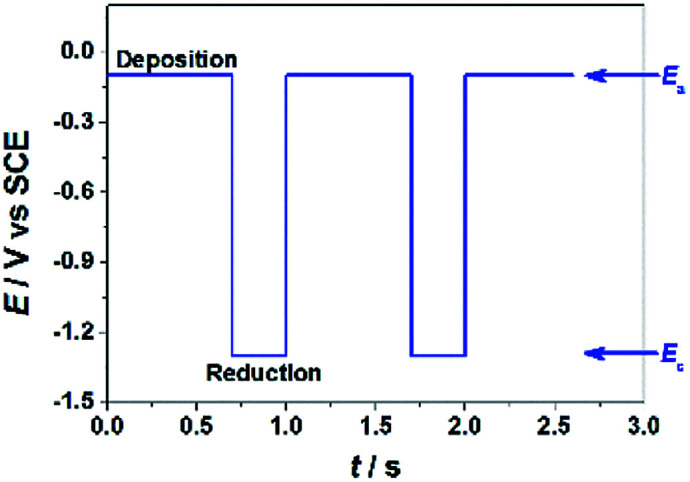
*E*–*t* diagram imposed during the electrodeposition preparation of ErGO-CP6 films.

GO colloids are negatively charged in the solution, while the surface charge of GO-CP6 is positively charged in the solution (Fig. S1, ESI†). When positive and negative potentials were applied on the GCE, respectively, GO and GO-CP6 could be spontaneously deposited onto the surface of GCE due to the strong electrostatic attraction. In accordance with the literature,^22^ the as-deposited GO can be electrochemically reduced at *E* = −1.1 V *vs.* SCE. Herein, pulse potentiostatic method was used to achieve the electrodeposition of ErGO and ErGO-CP6 films, in which 0.1 V and −0.1 V *vs.* SCE were used to deposit GO and GO-CP6 on GCE, respectively, followed by employing −1.3 V *vs.* SCE to electrochemically reduce the as-deposited GO and GO-CP6 to ErGO and ErGO-CP6. Fig. 2 illustrates the pulse process employed for the preparation of ErGO-CP6 films, and two alternative potentials *E*_a_ and *E*_c_ were used to prepare the ErGO-CP6 films on the surface of GCE. After the potential *E*_a_ is applied, GO-CP6 sheets were deposited onto the GCE surface during the period. When the potential *E*_c_ is employed, the GO-CP6 sheets close to the GCE surface start to react. Additionally, at the beginning of each new pulse, GO-CP6 sheets could be diffused to areas where they have been quickly consumed when applying *E*_c_. The electrodeposition process is controlled by a computer during the whole deposition so that such a procedure can give uniform thin films.

### Characterization of the ErGO/GCE and ErGO-CP6/GCE

**Fig. 3:**
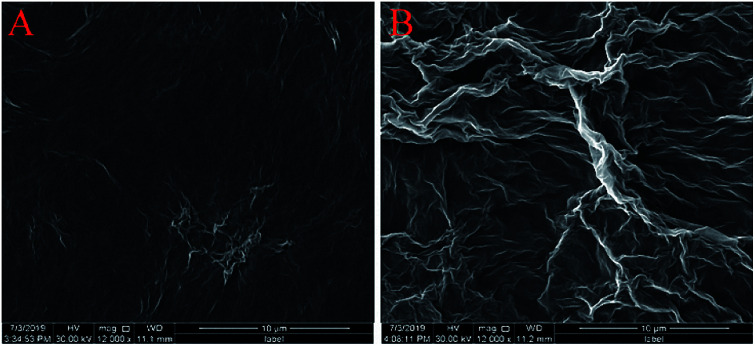
SEM images of ErGO films (A) and ErGO-CP6 films (B) modified GCE.

The surface morphologies of ErGO and ErGO-CP6 films electrodeposited on GCE were examined by SEM. Fig. 3A shows the SEM image of the ErGO films electrodeposited on the surface of GCE. As can be seen, the prepared ErGO films exhibit a curled morphology, a thin wrinkled paper-like structure, and distribute homogeneously on the GCE surface. In contrast, the prepared ErGO-CP6 films (Fig. 3B) possess more crumpled sheets closely associated with each other. Furthermore, aggregation barely occurs on the thin films, suggesting that the electrodeposition of ErGO-CP6 films on GCE by pulse potentiostatic method can obtain well-dispersed ErGO-CP6 films and prevent the aggregation.

**Fig. 4:**
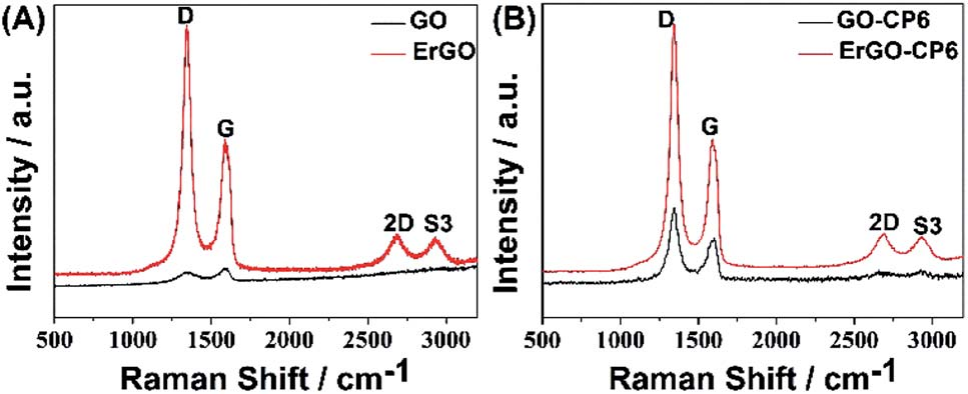
(A) Raman spectra of GO and ErGO. (B) Raman spectra of GO-CP6 and ErGO-CP6.

Raman spectroscopy is a widely used analytical method that is used to characterize the structural and electronic properties of graphene-based materials, involving disorder and defect structures.^41^ The D band at ∼1350 cm^−1^ corresponds to the breathing mode of *κ*-point phonons of A_1g_ symmetry, while the G band at ∼1575 cm^−1^ is usually assigned to the E_2g_ phonon of the sp^2^ carbon atoms.^42^Fig. 4 shows the Raman spectra of GO, ErGO, GO-CP6, and ErGO-CP6. The Raman spectrum of GO-CP6 (Fig. 4B) displays D and G bands at 1344 and 1587 cm^−1^, respectively, which is similar to those of GO prepared through the chemical oxidation of graphite (Fig. 4A). After GO and GO-CP6 were electrochemically reduced, the Raman spectra both display an increase in the intensities of D band compared to those of G band (Fig. 4A and B). The intensity ratio of the D band to the G band (*I*_D_/*I*_G_) of carbon products is generally used to evaluate the extent of disorder or defects which result from vacancies, distortion, and edges.^43,44^ The larger value of *I*_D_/*I*_G_ is an indication of smaller sp^2^ domains.^44^ As can be seen from Fig. 4A and B, after the electrodeposition, the *I*_D_/*I*_G_ ratios increase from 0.85 (for GO) to 1.81 (for ErGO) and 1.60 (for GO-CP6) to 1.84 (for ErGO-CP6), respectively, suggesting that smaller sp^2^ carbon domains are formed upon the electrochemical reduction of the GO and GO-CP6.^43^ The two weak and broad 2D bands at ∼2690 cm^−1^ also indicate disorder due to an out-of-plane vibrational mode, and the cooperation between D and G bands also gives rise to an S3 band near 2932 cm^−1^. The appearance of 2D and S3 bands at ErGO and ErGO-CP6 indicates that electrochemical reduction of GO and GO-CP6 can generate better graphitization compared to chemical reduction.^45^ The above results revealed that the electrochemical reduction of GO and GO-CP6 has indeed taken place, and their electrochemical reduction retained the sp^2^ hybridization of graphene's lattice.

**Fig. 5:**
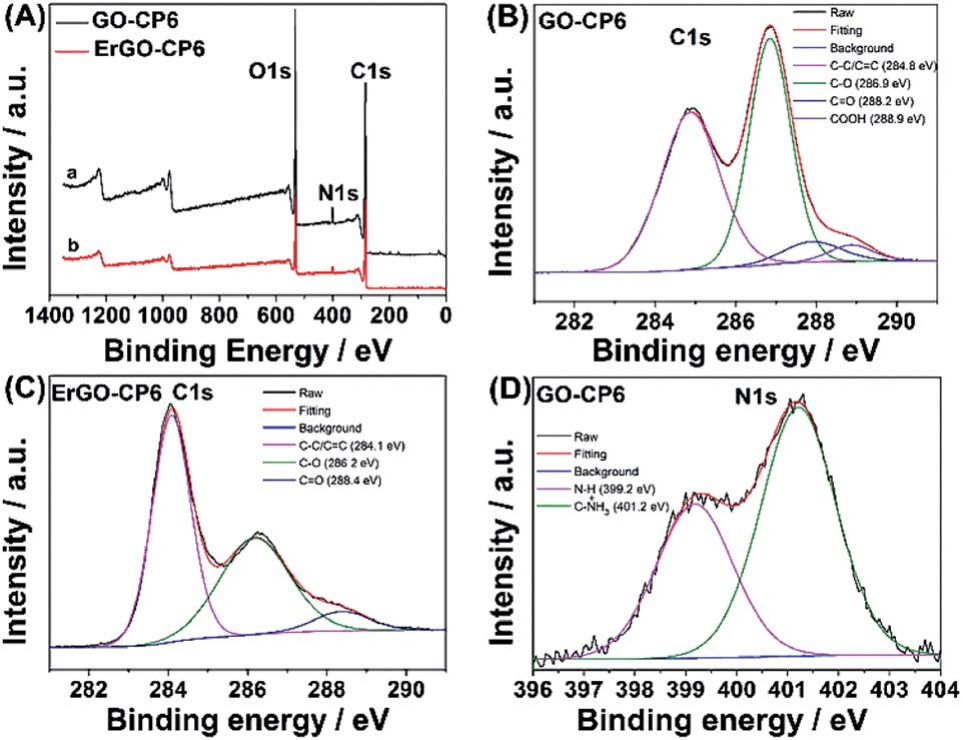
(A) XPS survey spectra obtained for (a) GO-CP6 and (b) ErGO-CP6. High resolution XPS C1s spectra obtained for (B) GO-CP6 and (C) ErGO-CP6. High resolution XPS N1s spectra obtained for (D) GO-CP6.

Further, XPS was used to confirm the electrochemical reduction of GO-CP6 to ErGO-CP6. Fig. 5A shows the XPS survey spectrum of GO-CP6 and ErGO-CP6. The GO-CP6 shows an asymmetric peak at 284 eV corresponding to the C 1s peak of sp^2^ carbon (curve a). The peak at 532 eV corresponds to the O 1s spectrum of various oxygen-containing groups. The intensity ratio of C/O peaks was calculated as 2.75. After the electrochemical reduction of GO-CP6 to ErGO-CP6, the XPS spectrum shows C 1s and O 1s peaks at 284 and 532 eV, respectively, with a variation in the C/O intensity ratio (curve b). The C/O intensity ratio was increased from 2.75 to 4.41 for CP6-modified GO after electrochemical reduction. This is due to the removal of oxygen-containing groups from the surface of GO. The electrochemical reduction of GO-CP6 to ErGO-CP6 was also confirmed by fitting C 1s spectra of GO-CP6 to ErGO-CP6 using Gaussian functions after background correction. Fig. 5B and C shows the C1s spectrum of GO-CP6 and ErGO-CP6 substrate. The C1s spectrum of GO-CP6 substrate shows four characteristic signal peaks at 284.8, 286.9, 288.2, and 288.9 eV, corresponding to C–C/C=C bond, C–O band, C=O band, and HO–C=O bond, respectively. On the other hand, the C1s spectrum of ErGO-CP6 substrate displays three peaks at 284.1, 286.2, and 288.4 eV, corresponding to C–C/C=C bond, C–O band, and C=O band, respectively. The intensities of the C–C/C=C peaks were remarkably increased, while the intensities of all C1s peaks of the carbons binding to oxygen, especially the peak of C–O, were dramatically decreased at the ErGO-CP6 substrate compared to the GO-CP6 substrate, indicating that electrochemical reduction of GO-CP6 to ErGO-CP6 removes the oxygen-containing groups on the electrode surface. Besides, there is no N–H group in amide in the N 1s XPS of GO-CP6 (Fig. 5D), indicating that CP6 molecules have been non-covalently attached to the surface of GO.

### Electrochemical characterization of the ErGO/GCE and ErGO-CP6/GCE

**Fig. 6:**
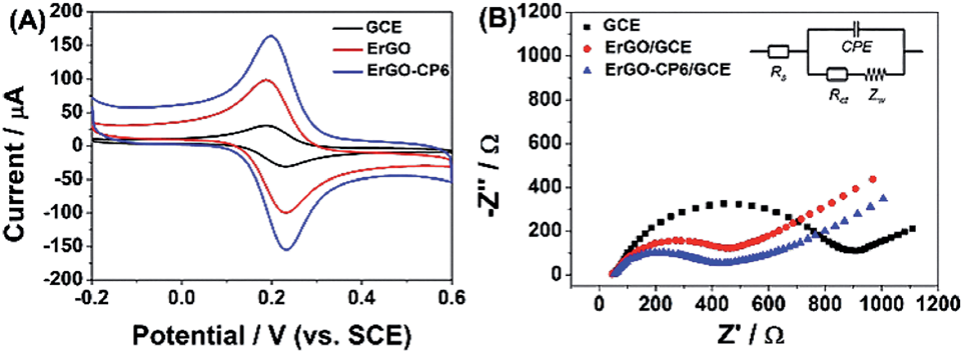
(A) CV curves of a bare GCE, ErGO/GCE and ErGO-CP6/GCE in solution containing 1.0 mM [Fe(CN)_6_]^3−/4−^ and 0.1 M KCl. Scan rate: 50 mV s^−1^. (B) Nyquist plots of a bare GCE, ErGO/GCE and ErGO-CP6/GCE in solution containing 1.0 mM [Fe(CN)_6_]^3−/4−^ and 0.1 M KCl.

The redox probe [Fe(CN)_6_]^3−/4−^, which is sensitive to surface chemistry of carbon-based electrodes,^46^ was commonly used to directly evaluate the charge transfer property of the modified electrodes. Fig. 6A displays the cyclic voltammograms (CVs) of 1.0 mM [Fe(CN)_6_]^3−/4−^ redox couple (1 : 1) on the surface of bare GCE, ErGO/GCE and ErGO-CP6/GCE in 0.1 M KCl solution at 50 mV s^−1^. It can be seen at the bare GCE, a pair of well-defined reversible redox peaks with the oxidation-to-reduction peak potential separation (Δ*E*_p_) of 81 mV present. While at the ErGO/GCE, both anodic and cathodic peak currents increase noticeably with the Δ*E*_p_ value decrease to 50 mV. The smaller Δ*E*_p_ observed at the ErGO/GCE exhibited a faster electron transfer process and coefficient^47,48^ compared to the bare GCE. Meanwhile, the enhanced current response of the ErGO/GCE toward [Fe(CN)_6_]^3−/4−^ indicates increased electrochemical active sites at the ErGO modified GCE surface, which is attributed to superior conductivity and large surface area of ErGO. After electrodeposition of ErGO-CP6 on GCE, the largest peak currents and the smallest Δ*E*_p_ (46 mV) of redox probe [Fe(CN)_6_]^3−/4−^ were observed. This is mainly attribute to the presence of positive charges on ErGO-CP6 which attract the negatively charged [Fe(CN)_6_]^3−/4−^ ions. At the same time, the effective surface areas of different electrodes were calculated by CV in the presence of 1.0 mM [Fe(CN)_6_]^3−^ solution containing 0.1 M KCl. The Randles–Sevcik formula of *i*_pa_ = 2.69 × 10^5^*n*^3/2^*AD*_o_^1/2^*c*_o_*v*^1/2^, where *i*_pa_ is the anodic peak current (A), *c*_o_ is the concentration of [Fe(CN)_6_]^3−^ (mol L^−1^), *v* is the scan rate (V s^−1^) and *A* is the surface area of the electrode (cm^2^), was used to calculate the surface area of the electrode (*n* = 1, *D*_o_ = 7.6 × 10^−6^ cm^2^ s^−1^).^49^ According to the calculation, the effective surface areas of bare GCE, ErGO/GCE and ErGO-CP6/GCE are 0.058, 0.541 and 0.633 cm^2^, respectively. Therefore, it is clear that the highest electroactive surface was obtained on ErGO-CP6/GCE. Such results suggest that the properties of the ErGO-CP6 films prepared by pulsed electrodeposition method are superior to those of ErGO films in increasing the active surface area of the modified electrode and accelerating the electron transfer rate.

The Nyquist plots of bare GCE, ErGO/GCE and ErGO-CP6/GCE electrode surfaces in 1.0 mM [Fe(CN)_6_]^3−/4−^ containing 0.1 M KCl (Fig. 6B) exhibited a significant difference. The values of charge transfer resistance (*R*_ct_) of the bare GCE, ErGO/GCE and ErGO-CP6/GCE were estimated to be 0.96, 0.57 and 0.39 kΩ, respectively. Therefore, ErGO-CP6/GCE has more efficient conductivity compared with bare GCE and ErGO/GCE. This is consistent with the results obtained with the electroactive surface area.

### Electrochemical performance of ErGO-CP6 modified electrode

**Fig. 7:**
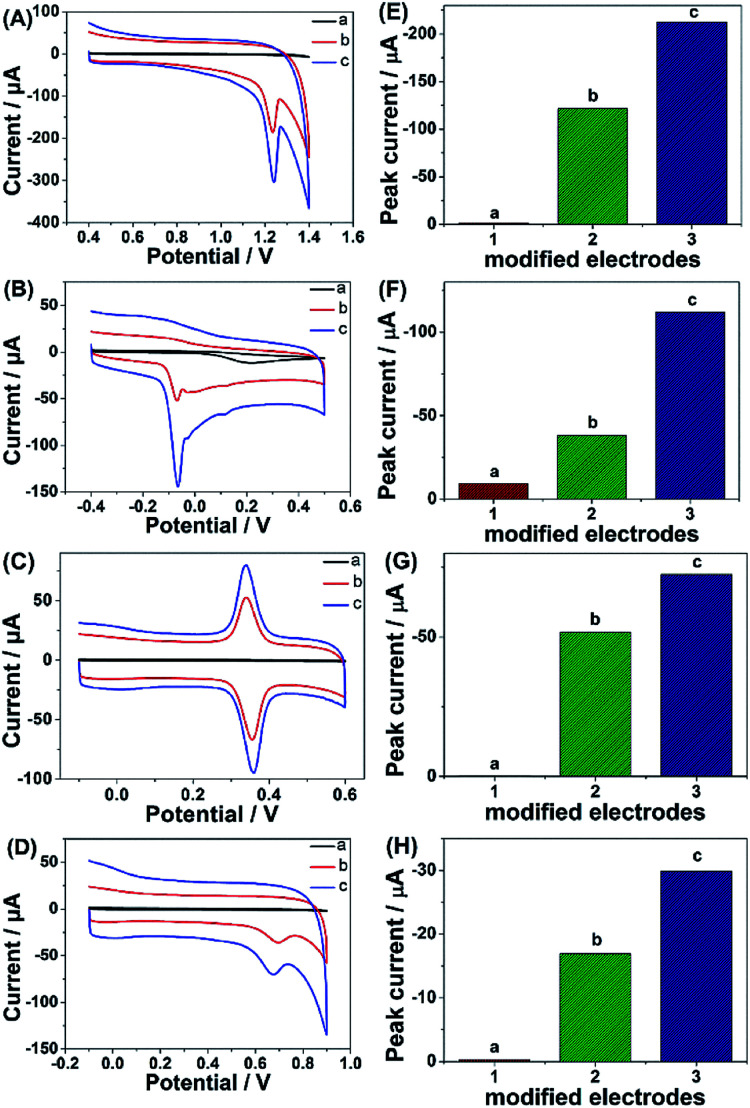
CVs and peak current of CVs of (A and E) 5 μM TP, (B and F) 5 μM AA, (C and G) 5 μM APAP and (D and H) 5 μM FA in 0.2 M PBS (pH 6.8) on (a) bare GCE, (b) ErGO/GCE and (c) ErGO-CP6/GCE. Scan rate: 50 mV s^−1^.

Given the above discussion, it can be demonstrated that CP6-modified graphene nanocomposite films had been prepared on GCE by pulsed electrodeposition, which could not only improve the stability and dispersion of graphene but also enhance sensitivity for detecting some important biological molecules through supramolecular host–guest complex formation between CP6 and the guest molecules that fit spatially within CP6 cavities. To confirm this conception (Scheme 1), the electrochemical behaviors of four electroactive biomolecules [theophylline (TP), ascorbic acid (AA), acetaminophen (APAP), and folic acid (FA)] were investigated. CVs and peak currents of the above four guests on (a) GCE, (b) ErGO/GCE and (c) ErGO-CP6/GCE are shown in Fig. 7A–D and E–H, respectively. As shown in Fig. 7A–D (curve a), very weak redox peaks currents on bare GCE were observed for four guests. While, there were increases in the oxidation peak currents of four guests at ErGO/GCE compared to the currents at the bare GCE (Fig. 7A–D, curve b), which may be ascribed to excellent conductivity and large surface area of ErGO arising from its specific structure. Much to our excitement, on the ErGO-CP6/GCE (Fig. 7A–D, curve c), all the peak currents were remarkably increased and were approximately 1.5–3.0 times as much as those on ErGO/GCE. Meanwhile, ErGO-CP6/GCE for four guests also showed highest current densities in two kinds of modified electrodes (Table 1). The high sensitivity for ErGO-CP6/GCE can be explained: (a) CP6 immobilized on ErGO with supramolecular enrichment ability has a high affinity to all the examined analytes to form supramolecular host–guest complexes (the association constant; see Table S1 in the ESI†). The host–guest interactions between the CP6 and the analytes can further improve the accumulation effect of ErGO-CP6/GCE and therefore increase the analytes concentration on the surface of the modified electrode, which resulted in the remarkable enhancement in the peak current as compared with ErGO/GCE. (b) CP6 functionalized electrochemically reduced graphene oxide shows important properties such as high electrode area and higher conductivity. Therefore, the synergetic effect between CP6 and ErGO are probably responsible for the enhanced electrochemical performance in the detection of TP, AA, APAP and FA on ErGO-CP6/GCE.

**Table 1:** Current densities of CVs of 5 μM TP, AA, APAP, and FA in 0.2 M PBS (pH 6.8) on ErGO/GCE and ErGO-CP6/GCE

Guest molecules	Current densities (mA mM^−1^ L cm^−2^)	
	ErGO/GCE	ErGO-CP6/GCE
TP	44.90 ± 0.12	79.57 ± 0.45
AA	14.10 ± 0.14	41.40 ± 0.15
APAP	19.10 ± 0.15	27.60 ± 0.20
FA	6.30 ± 0.24	11.10 ± 0.15

**Fig. 8:**
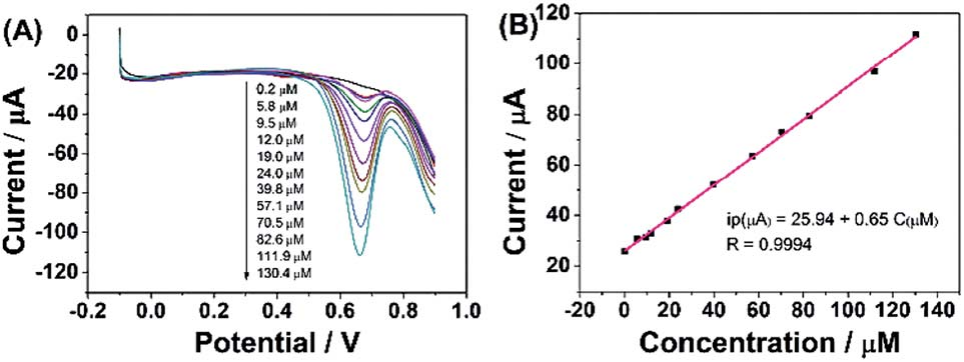
(A) DPV response for the different concentrations of FA on ErGO-CP6/GCE in 0.2 M PBS (pH 6.8): pulse period, 0.2 s; amplitude, 50 mV. (B) The calibration curve of FA.

According to the above discussion, ErGO-CP6 is an excellent electrode material for improving the electrochemical response for different analytes. To evaluate the sensing performance of ErGO-CP6 toward certain substances, FA (FA, being the coenzyme that controls the generation of ferroheme) was chosen as a representative analyte. Fig. 8A displays the differential pulse voltammetric (DPV) response of ErGO-CP6/GCE for different concentration additions of FA. Under the optimized conditions, the DPV response of FA is linearly proportional to the concentration within 0.2–130.4 μM, and the corresponding linear regression equation can be expressed as *I*_pa_ (μA) = 25.94 + 0.65*C*_FA_ (μM) with the correlation coefficient (*R*^2^) of 0.9994. The detection limit of FA was estimated to be 40 nM based on S/N = 3 (Fig. 8B), which is better than or comparable with that of previously reported FA sensors, as shown in Table 2. In addition, the linear response range obtained by ErGO-CP6/GCE is comparable with previously reported FA sensors. The comparative results clearly reveal that the ErGO-CP6/GCE exhibits an excellent electrochemical performance toward the target molecule. Furthermore, the present method of fabrication of ErGO-CP6/GCE is easy, simple and time-saving when compared to other carbon-based nanomaterials modified electrode. Therefore, based on a pulsed electrodeposition technique, ErGO-CP6/GCE can be used as a promising electrode material for sensitive detection of a wide variety of electroactive compounds.

**Table 2:** Comparison with other modified electrodes in the literatures for the detection of FA using DPV

Modified electrode	Linear range (μM)	Detection limit (nM)	Ref.
MoS_2_-rGO/GCE	0.01–100	10	51
Methylene blue/rGO/GCE	4–167	500	52
Co_3_O_4_/rGO/cetyltrimethyl ammonium bromide/CPE	300–3200	19	53
Graphene/Pt/CPE	1–1000	1.0	54
Poly(5-amino-2-mercapto-1,3,4-thiadiazole)/GCE	0.1–800	0.23	55
1-Octyl-3-methylimidazolium hexafluorophosphate-single-walled carbon nanotube/GCE	0.002–4.0	1.0	56
Poly(3-amino-5-mercapto-1,2,4-triazole)/GCE	20–180	250	57
ErGO-CP6/GCE	0.2–130.4	40	This work

On the other hand, as can be seen from Fig. S3 in the ESI,† CP6 could be oxidized and the anodic peak potential was about 0.2 V. Therefore, the electrochemical reactions of ErGO-CP6/GCE in blank PBS buffer within the corresponding experimental potential window for TP (Fig. S4A, ESI†), AA (Fig. S4B, ESI†), APAP (Fig. S4C, ESI†), and FA (Fig. S4D, ESI†) were investigated in order to see if there was the interference anodic peak of CP6. As for APAP, Fig. S4C† displays CVs of 0.2 M blank PBS (curve a) and 5 μM APAP in 0.2 M PBS (curve b) at ErGO-CP6/GCE. As it can be seen, there was no electrochemical response of CP6 in the −0.1 to 0.6 V in 0.2 M blank PBS, suggesting that the ErGO-CP6 coated electrode is inert in this electrochemical window. However, high oxidation peak current of APAP was observed when 5 μM APAP was present in the PBS. The result suggested that the peak current value of APAP cannot be affected by CP6. Similar results as shown in Fig. S4A, B and D† have also been achieved for TP, AA, and FA.

### Interference study

**Fig. 9:**
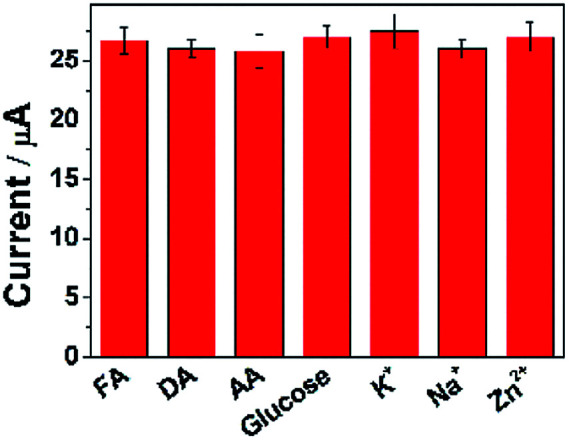
The current response of ErGO-CP6/GCE in solution containing 2 μM FA in the absence and presence of 100-fold of DA, AA, glucose, K^+^, Na^+^, and Zn^2+^, respectively, using DPV and keeping all the parameters constant.

To ascertain the selectivity of ErGO-CP6/GCE to the substrates, FA was chose as a representative analyte and DPV was performed for the electro-oxidation of FA (concentration: 2 μM) along with 100-fold concentration of other possible co-existing inorganic metal ions (K^+^, Na^+^, Zn^2+^) and interfering biomolecules (DA, AA, and glucose). The observed results (Fig. 9) clearly indicated that the oxidation peak current of FA was not affected even in the presence of excess concentration of the interfering ions and biomolecules, which clearly confirmed that the ErGO-CP6/GCE possesses satisfactory selectivity to FA. These results can be expounded from the interactions between CP6 and FA, *i.e.*, electrostatic interaction. As for electrostatic interaction, because CP6 has a positive charge and FA has a negative charge, there is an intense electrostatic interaction between CP6 and FA, which results in the strong interactions between CP6 and FA. However, DA carry with positive charge, so the similar result cannot be obtained. In the case of AA, as can be seen from Table S1 in the ESI,† the binding constant of FA with CP6 is 10-fold higher than that of AA with CP6. Therefore, negligible interference has been found.

### Stability and reproducibility of ErGO-CP6/GCE

**Fig. 10:**
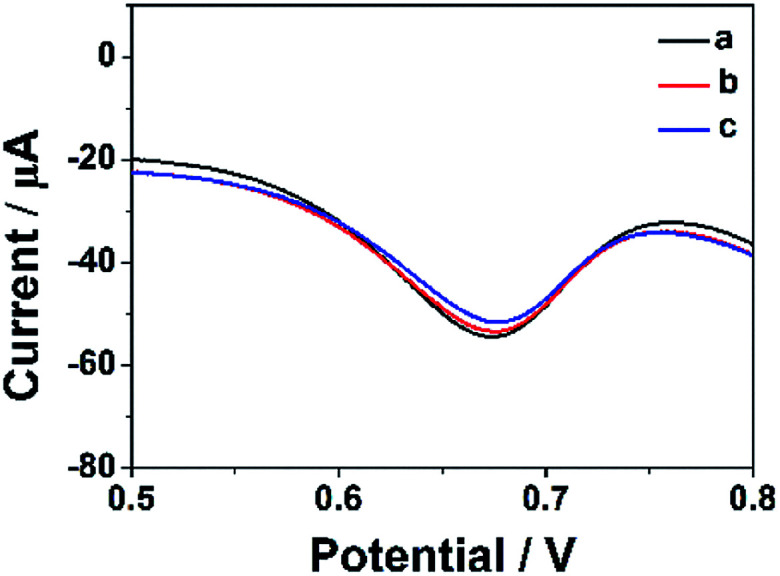
DPV response for the oxidation of 40 μM FA at the ErGO-CP6/GCE in 0.2 M pH 6.8 PBS solution at different days: (a) 1, (b) 7, and (c) 14.

In order to estimate the stability of the ErGO-CP6/GCE, the oxidation of FA was recorded for two weeks. Fig. 10 displays the DPVs obtained for 40 μM FA at ErGO-CP6/GCE in 0.2 M PBS solution (pH 6.8) from the first day to fourteenth day (curves a–c). As can be seen from Fig. 10, the FA oxidation peak current remains 94.5% of its initial current after 14 days' storage, indicating the ErGO-CP6/GCE has good stability. Further, to check the reproducibility of the results, five different GC electrodes were prepared with ErGO-CP6 under similar conditions. Their peak potential and current response toward the oxidation of FA were the same at all five electrodes. All these results showed that ErGO-CP6/GCE was very stable and reproducible.

### Analysis of real samples

To evaluate the practical applicability of the proposed ErGO-CP6/GCE, the developed method was utilized to determination of FA in actual samples. FA tablets (0.40 mg per pill) were purchased from a local pharmacy (Zhengzhou, China). FA tablets were grounded up and dissolved in 10 mL of 0.2 M PBS (pH 6.8), and the standard addition method was used for the accurate determination. An average recovery of 98.5% for FA was detected in FA tablets. This result suggests that the proposed ErGO-CP6/GCE is highly suitable for the determination of FA in real samples.

## Experimental section

### Preparation of GO-CP6 composites

GO was prepared from natural graphite powder by a modified Hummer's method,^50^ and CP6 was prepared according to our previously published procedure.^38^ A GO-CP6 composite was prepared as follows: CP6 (6 mg) and GO (6 mg) were dissolved in 10 mL of doubly distilled water (DDW) by sonication for 10 min, and then the mixture reacted for 12 h at room temperature under continuous stirring. The black dispersion was separated by centrifuging at 18 000 rpm for 20 min, then rinsed with DDW three times thoroughly, and dried under vacuum to obtain GO-CP6 composite. The GO-CP6 powder which can be easily dispersed in a 0.2 M pH 6.8 PBS by ultrasonication again was obtained by freeze drying for further characterization.

### Pulsed electrodeposition preparation of ErGO and ErGO-CP6 films onto GCE

Prior to use, the GCE surface was successively polished with 0.3 and 0.05 μm Al_2_O_3_ powder and washed thoroughly with DDW between each polishing step, and then the polished GCE was sonicated in ethanol and DDW for 2 min prior to each experiment, then dried under N_2_ blowing. After drying, the cleaned GCE was immersed in the aforementioned PBS (pH 6.8) containing 0.8 mg mL^−1^ GO-CP6, and the GO-CP6 was electrodeposited onto the GCE by pulse potentiostatic method under constant stirring at room temperature. The optimum pulse electrodeposition parameters were set as follows: anodic potential (*E*_a_), −0.1 V; cathodic potential (*E*_c_), −1.3 V; anodic pulse duration time (*t*_a_), 0.6 s; cathodic pulse duration time (*t*_c_), 0.3 s; the total experimental time (*t*_exp_), 150 s. The reduction time (*t*_re_) can be calculated from the following equation: *t*_re_ = *t*_exp_ × *t*_c_/(*t*_c_ + *t*_a_). The optimal electrodeposition parameters (*t*_a_, *t*_c_ and *t*_re_) were described in the ESI.† After electrodeposition, the ErGO-CP6/GCE was thoroughly washed with DDW and then kept under ambient conditions prior to use. For comparison purposes, we also prepared ErGO/GCE through the similar pulse potentiostatic method except that the *E*_a_ = 0.1 V was used to deposit GO on GCE.

## Conclusions

In summary, we have developed a simple, rapid and green pulsed electrodeposition method for the preparation of ErGO-CP6 films on GCE surface. The prepared ErGO-CP6 films were characterized by SEM, Raman spectroscopy and XPS. SEM image confirms that ErGO-CP6 could form a homogeneous film when electrodeposited on the surface of GCE. Raman spectrum displays the increase in the intensity ratio of D band compared to that of G band after GO-CP6 was electrochemically reduced to ErGO-CP6, suggesting that the graphene backbone was retained during the reduction process. XPS results confirm the removal of oxygen-containing functional groups present on the GO-CP6 surface after electrochemical reduction. More significantly, due to the good electrical properties of graphene and the supramolecular enrichment capabilities of CP6, the ErGO-CP6 films at the modified electrode could exhibit much higher electrochemical response toward TP, AA, APAP, and FA than those of ErGO/GCE and bare GCE. Under optimal conditions, the detection limit of FA was 40 nM. It is predicted that the directly electrodeposited reduced graphene oxide-cationic pillar[6]arene composite films could be widely used as a promising platform for analytical sensing owing to their good supramolecular enrichment capabilities and excellent electrochemical responses.

## Conflicts of interest

There are no conflicts to declare.

## Supplementary Material

RA-010-D0RA03138K-s001
