# Correction: Direct electrodeposition of cationic pillar[6]arene-modified graphene oxide composite films and their host–guest inclusions for enhanced electrochemical performance

**DOI:** 10.1039/d0ra90132f

**Published:** 2020-12-18

**Authors:** Qunpeng Duan, Lijie Wang, Fei Wang, Hongsong Zhang, Kui Lu

**Affiliations:** School of Materials and Chemical Engineering, Henan University of Engineering Zhengzhou 450006 China qpduan@haue.edu.cn; School of Chemical Engineering and Food Science, Zhengzhou Institute of Technology Zhengzhou 450044 China luckyluke@haue.edu.cn

## Abstract

Correction for ‘Direct electrodeposition of cationic pillar[6]arene-modified graphene oxide composite films and their host–guest inclusions for enhanced electrochemical performance’ by Qunpeng Duan *et al.*, *RSC Adv.*, 2020, **10**, 21954–21962, DOI: 10.1039/D0RA03138K.

The authors regret omitting a citation of their related paper published in *Frontiers in Chemistry*: ‘Facile one-step electrodeposition preparation of cationic pillar[6]arene-modified graphene films on glassy carbon electrodes for enhanced electrochemical performance’ (DOI: 10.3389/fchem.2020.00430) shown as ref. 1 here. The citation should appear as ref. 58 in the original article.^1^

The authors regret that it was not clear in the original article that the ErGO-CP6/GCE film had been previously reported by them in their related *Frontiers in Chemistry* paper^1^ and therefore the sentence at the start of paragraph 3 on page 2 ‘In this work, we report for the first time preparation of CP6 functionalized graphene films on glassy carbon electrode (GCE) directly from GO-CP6 dispersions by facile one-step pulsed electrodeposition technique (Scheme 1).’ should be ‘In this work, we report the preparation of CP6 functionalized graphene films on glassy carbon electrode (GCE) directly from GO-CP6 dispersions by facile one-step pulsed electrodeposition technique (Scheme 1), which was previously reported by us.^58^’.

The authors also wish to clarify the differences between this *RSC Advances* paper and the *Frontiers in Chemistry* paper.^1^ The papers use different guests molecules and different optimum pulse electrodeposition parameters and the *RSC Advances* paper reports an improvement in electrochemical performance with additional characterisation, stability studies and the analysis of real samples which are not reported in the *Frontiers in Chemistry* paper.^1^

The appropriate figure captions have been updated to reflect the data reproduced from the *Frontiers in Chemistry* paper.^1^

Scheme 1 Schematic illustration for the pulsed electrodeposition preparation of ErGO and ErGO-CP6 films on the surface of GCE and sensing the guest molecules by an electrochemical strategy. Reproduced with permission from ref. 1. Copyright 2020 Frontiers.

Fig. 1 Characterization of materials. FTIR spectra (A), UV-vis absorption spectra (B), TGA curves of CP6, GO-CP6, and GO (C), and XPS survey spectra of GO and GO-CP6 (D). The data in (a, c and d) have been reproduced with permission from ref. 1. Copyright 2020 Frontiers.

Fig. 4 (A) Raman spectra of GO and ErGO. (B) Raman spectra of GOCP6 and ErGO-CP6. Reproduced with permission from ref. 1. Copyright 2020 Frontiers.

## Supplementary Material
