# Supplementary material for: Direct electrodeposition of cationic pillar[6]arene-modified graphene oxide composite films and their host–guest inclusions for enhanced electrochemical performance
Source: RSC Adv. 2020 Jun 9;10(37):21954–62. doi: 10.1039/d0ra03138k (PMC9054522; doi:10.1039/d0ra03138k)
Supplement: RA-010-D0RA03138K-s001 [file RA-010-D0RA03138K-s001.pdf]

Electronic Supplementary Material (ESI) for RSC Advances.
This journal is © The Royal Society of Chemistry 2020

Electronic Supplementary Information

Direct Electrodeposition of Cationic Pillar[6]arene-Modified Graphene Oxide Composite Films and Their Host-Guest Inclusions for Enhanced Electrochemical Performance

Qunpeng Duan,*[a] Lijie Wang,[a] Fei Wang,[a] Hongsong Zhang[a] and Kui Lu*[a,b]

[a]*School of Materials and Chemical Engineering, Henan University of Engineering, Zhengzhou, 450006, China.*

[b]*School of Chemical Engineering and Food Science, Zhengzhou Institute of Technology, Zhengzhou, 450044, China.*

Supporting Information (9 pages)

1. *Materials and methods* S2
2. *Zeta potentials of GO and GO-CP6* S3
3. *Optimal parameters of electrodeposition* S3
4. *CV curves of CP6 and ErGO-CP6/GCE* S4
5. *Determination of the association constants between substrates (**TP, AA, APAP, FA**) and **CP6*** S5
6. *Reference* S8

1. *Materials and methods*

Graphite was obtained from XFNANO Materials Tech Co., Ltd (Nanjing, China). TP, AA, APAP, and FA were purchased from Adamas-beta Ltd. All other reagents were all analytically pure, used as supplied without further purification. Doubly distilled water (DDW) was used for preparing all solutions. Fourier transform infrared (FTIR) spectroscopy measurements were conducted on a Thermo Fisher Nicolet 6700. UV-vis spectroscopy data were collected by a Shimadzu UV-3600 spectrophotometer (UV-3600, Shimadzu, Japan). Thermogravimetric analysis (TGA) was performed using NETZSCH STA449F3 thermogravimetric analyzer at a heating rate of 10 $°C·min^{-1}$ under nitrogen atmosphere from 30 to 800 °C. Raman spectra were recorded using an inVia Reflex Raman spectrometer (Renishaw Co., England). A Quanta-250 scanning electron microscope (SEM) (FEI, Czech) was used for imaging. X-ray photoelectron spectroscopy (XPS) data were collected with Thermo Fisher Scientific ESCALAB-250XI spectrometer. Al K alpha radiation was used as X-ray source (1486.6 eV). Zeta potential measurements were conducted on a Malvern Zetasizer Nano series. All fluorescence titration experiments were conducted on a Cary Eclipse fluorescence spectrophotometer (Agilent, Australia) at room temperature. Electrochemical experiments were carried out using a CHI 650A electrochemical analyzer (CHI Instrument, China) and RST5000 electrochemical workstation (Zhengzhou Shiruisi Technology, China). A conventional three-electrode system was employed, where a saturated calomel electrode (SCE) served as the reference electrode, a platinum (Pt) wire electrode as the auxiliary electrode, and the modified GCE (d = 3.0 mm) as the working electrode. The electrochemical impedance measurements were measured with an Autolab/PG30 electrochemical analyzer system (CIMPS-2 Zahner) and were performed in a 0.1 M KCl solution containing 1.0 mM $K_3[Fe(CN)_6]$+5mM $K_4[Fe(CN)_6]$ (1:1) at room temperature. The interfacial charge-transfer resistances for different modified surface were determined by EIS in the frequency range from 0.1 Hz to 1 MHz with a perturbation signal of 5 mV. All pH values were measured with a PHS-3C digital pH meter (Shanghai Leici Instrument Factory, Shanghai, China), which was calibrated daily at 25 °C.

2. Zeta potentials of GO and GO-CP6

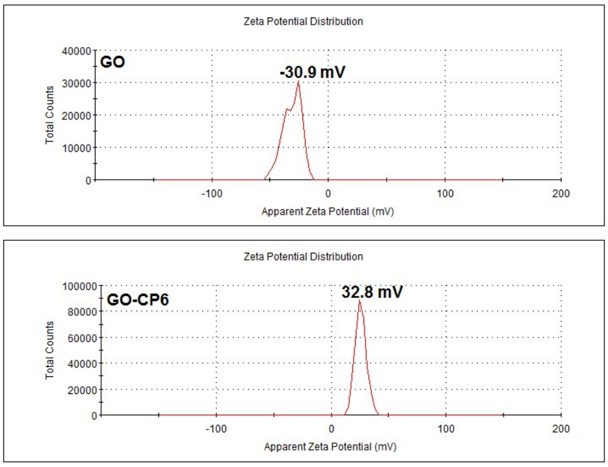

Figure S1. Zeta potentials of GO and GO-CP6.

3. Optimal parameters of electrodeposition

Figure S1A shows the CVs of redox probe on the surface of ErGO-CP6/GCE fabricated by pulse potentiostatic method under various t_{re} while keeping t_c/t_a and t_a. No saturation of the peak current is observed when the t_{re} increased from 1 to 80 s, while the ΔE_p continuously became smaller. The narrowed ΔE_p of $[Fe(CN)_6]^{3-/4-}$ suggested the improved electron transfer kinetics by ERGO films. Though the increase of t_{re} can increase the response signal, it also results in the low stability of the modified electrodes because the thick ErGO-CP6 films have weak adhesion to the electrode interface. So in the following experiments, t_{re} = 50 s was chosen as electrodeposition parameters. Then, the influence of t_c/t_a was investigated. Figure S1B shows the relationship of the average currents of redox probe with pulse electrodeposition of ErGO-CP6 film on GCE by varying the t_a/t_c as 3, 2, 1, 0.5, 0.33 while keeping t_{re} = 50 s and t_a =0.6 s. It is clear that the value of t_a/t_c in the range of 2-1, the currents reached steady and maximum, so t_a/t_c =2 was adopted. In the same way, t_c =0.3 s was used to construct the ErGO-CP6/GCE.

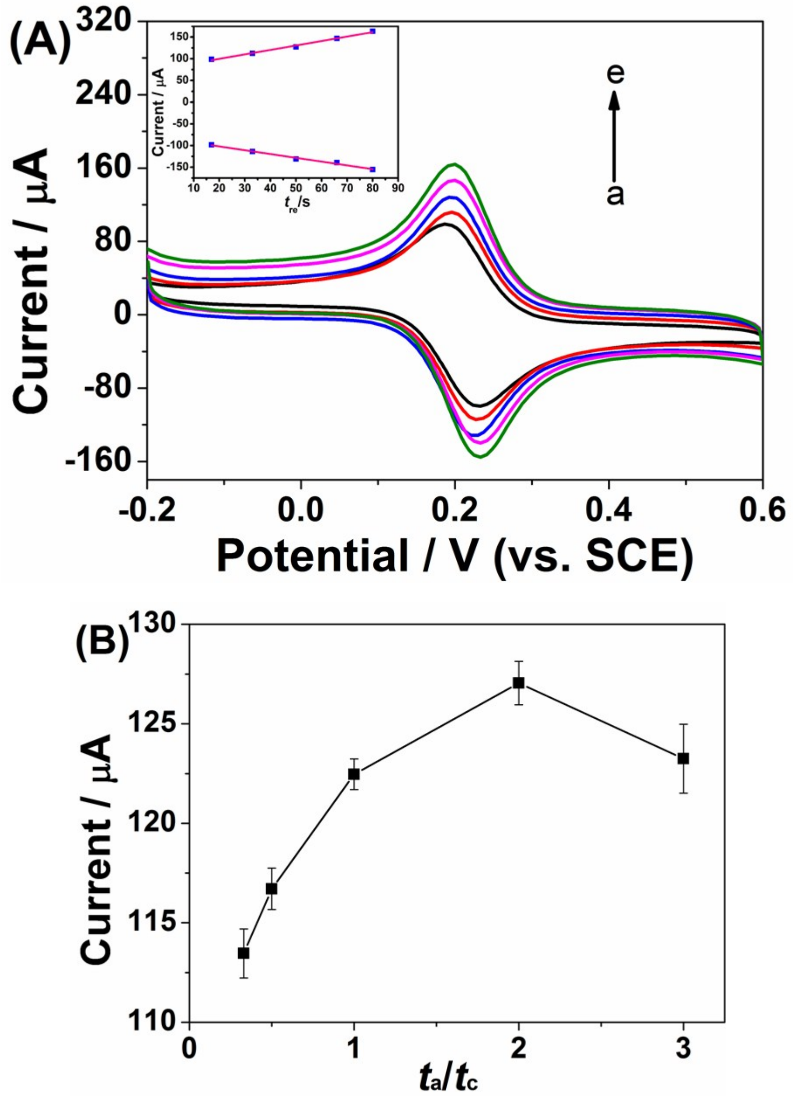

Figure S2. (A) CV curves of ErGO-CP6/GCE in solution containing 1.0 mM $[Fe(CN)_6]^{3-/4-}$ and 0.1 M KCl under various t_{re}, v = 100 mV·s^{-1}, t_a/t_c=2 and t_c=0.3s; the inset is the dependence average peak currents on t_{re}; (B) Corresponding average currents of $[Fe(CN)_6]^{3-/4-}$ on the ErGO-CP6 modified GCE under various t_a/t_c, t_{re} =50 s and t_c =0.3 s.

4. CV curves of CP6 and ErGO-CP6/GCE

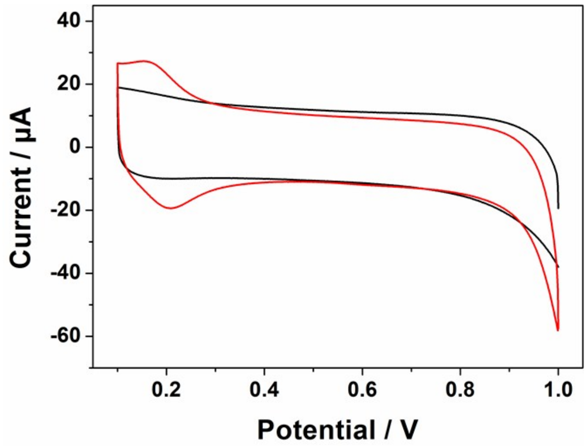

Figure S3. The voltammograms of the background and the solution containing **CP6**: 25 ^{o}C, 50 $mV \cdot s^{-1}$, 5 × 10^{-4} M **CP6** + 0.2 M PBS (pH = 7.00), black line: background; red line: solution containing CP6.

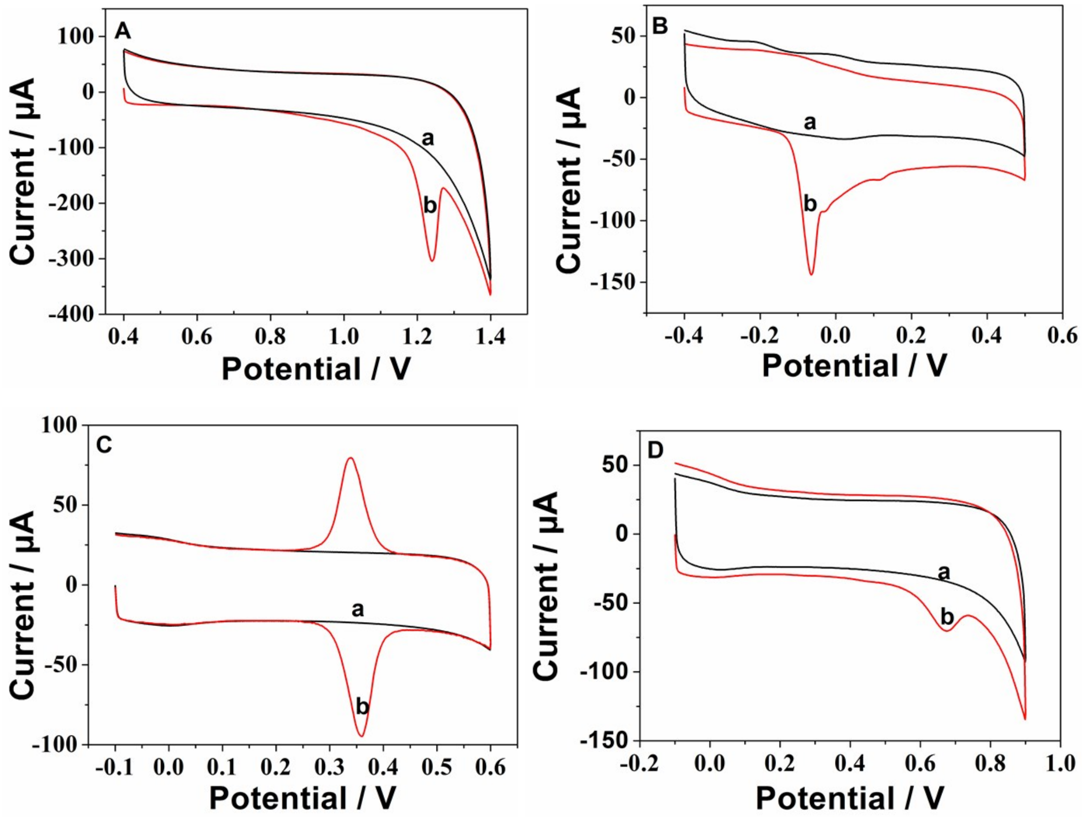

Figure S4. CVs of 0.2 M blank PBS (curve a) and (A) 5 μM TP, (B) 5 μM AA, (C) 5 μM APAP, and (D) 5 μM FA in 0.2 M PBS (curve b) at ErGO-CP6/GCE.

*5. Determination of the association constants between substrates **(TP, AA, APAP, FA)** and **CP6***

*5.1 Job plot for **CP6⊃TP/AA/APAP/FA***

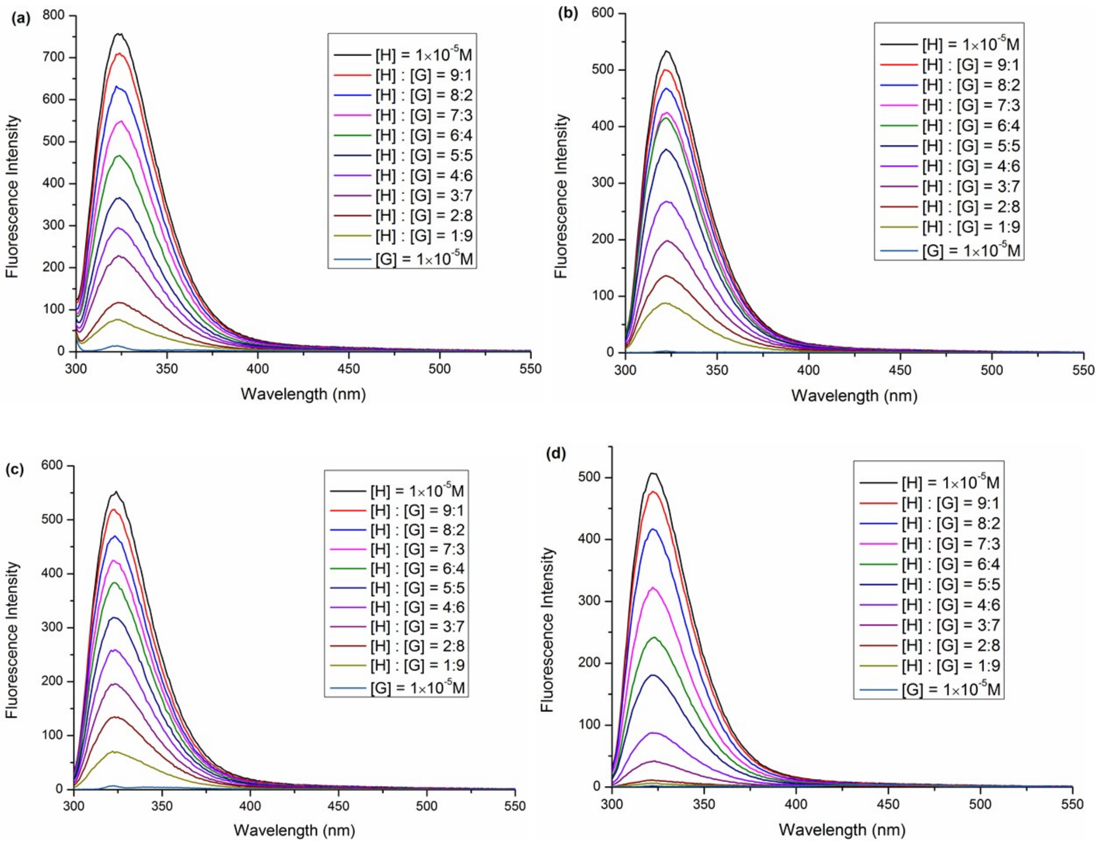

Figure S5. (a-d) Fluorescence spectra of the mixture of **CP6** and substrates (**TP**, **AA**, **APAP**, **FA**) in water at different molar ratios while [**CP6**] + [substrates] = 1.0 × 10^{-5} M, respectively.

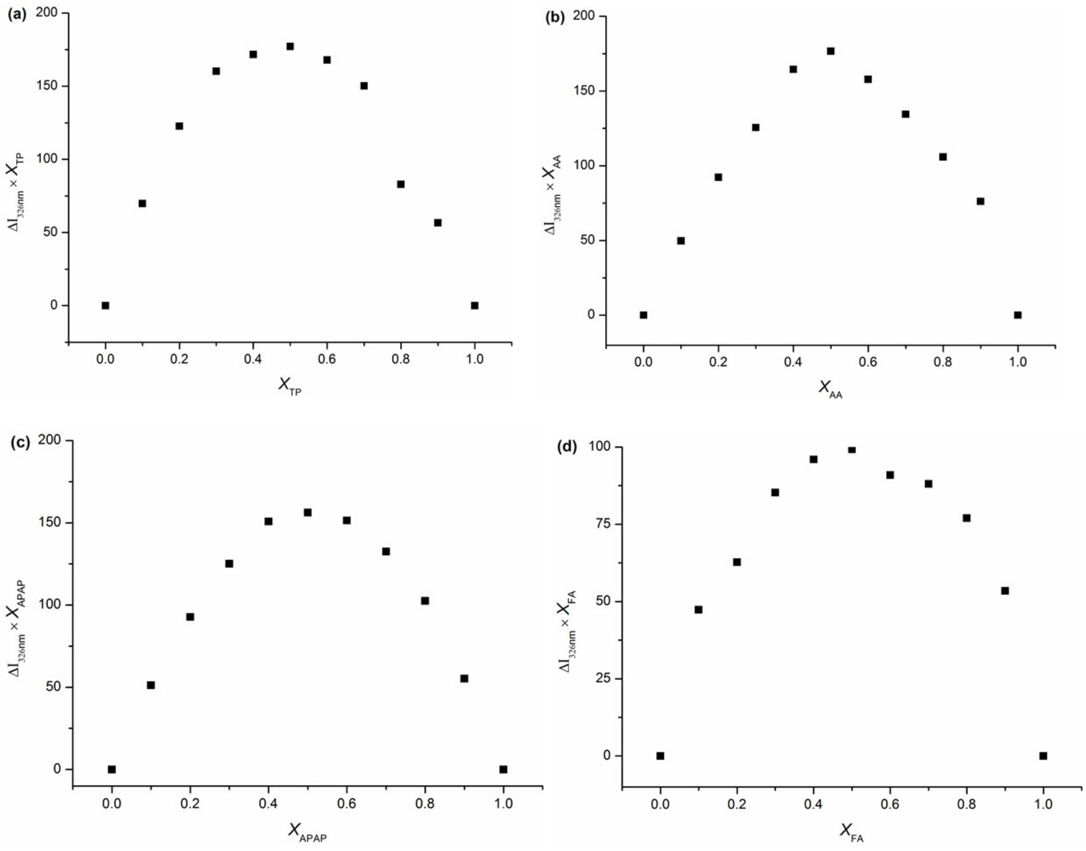

Figure S6. Job plot showing the 1:1 stoichiometry of the complex between **CP6** and substrates (**TP**, **AA**, **APAP**, **FA**) by plotting the difference in fluorescent emission intensity at $\lambda_{emission}$ = 326 nm ($\lambda_{excitation}$ = 290 nm) against the mole fraction of substrates at an invariant total concentration of 0.01 mM in aqueous solution, respectively.

5.2 Association constants of ***CP6⊃ TP/AA/APAP/FA***

To determine the association constants for the complexation between **CP6** and substrates (**TP, AA, APAP, FA**), fluorescence titration experiments were carried out in solutions which had a constant concentration of **CP6** (1.0×10^{-5} M) and varying concentrations of substrates. By a non-linear curve-fitting method, the association constants (K_a) of **CP6⊃TP/AA/APAP/FA** were estimated.

The non-linear curve-fittings were based on the equation:

$$\Delta F = (\Delta F_{\infty}/[H]_0)\ (0.5[G]_0 + 0.5([H]_0+1/K_a)-(0.5\ ([G]_0^2+(2[G]_0(1/K_a - [H]_0)) + (1/K_a + [H]_0)^2)^{0.5}))$$

(eq. 1)

Where ΔF is the fluorescence intensity changes at 326 nm at $[H]_0$, ΔF_{∞} is the fluorescence intensity changes at 326 nm when **CP6** is completely complexed, $[G]_0$ is the initial concentration of substrates (**TP, AA, APAP, FA**), and $[H]_0$ is the fixed initial concentration of **CP6**.[S1]

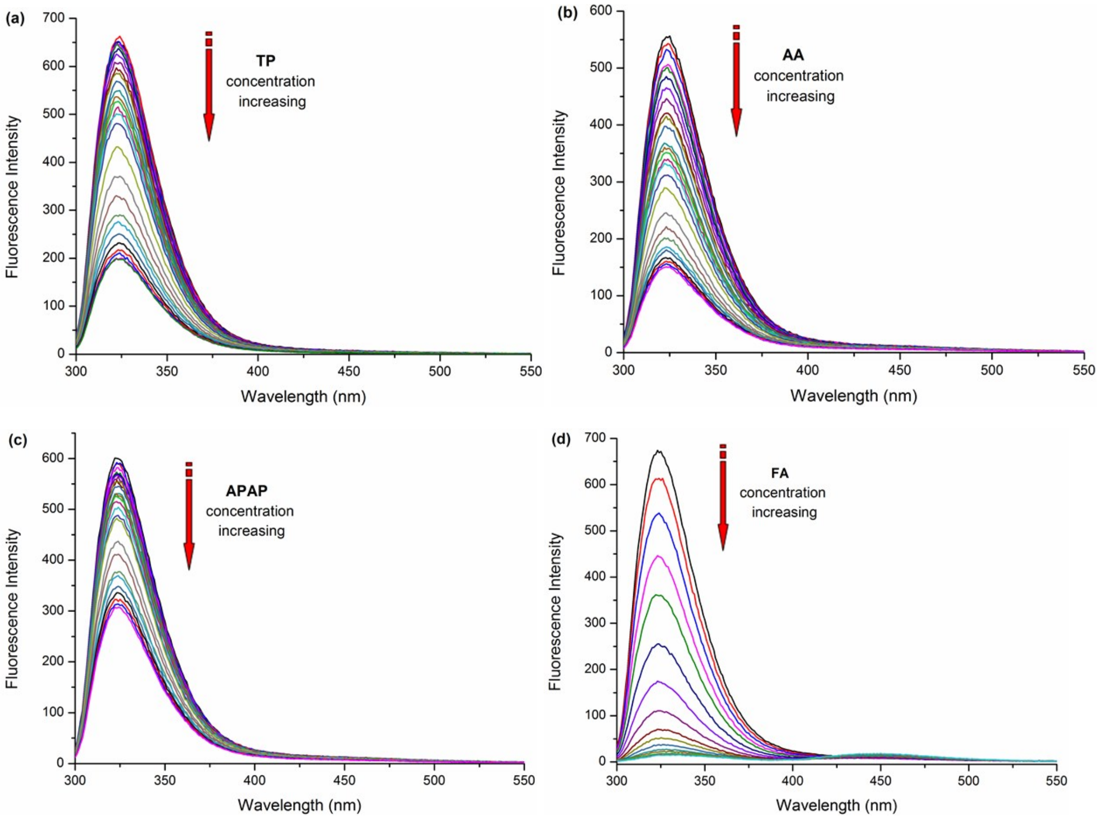

Figure S7. (a-j) Fluorescence spectra of **CP6** (1.0×10^{-5} M) upon addition of substrates (**TP, AA, APAP, FA**) (0-5.43×10^{-4} M) in aqueous solution (excited at 290 nm) at room temperature, respectively.

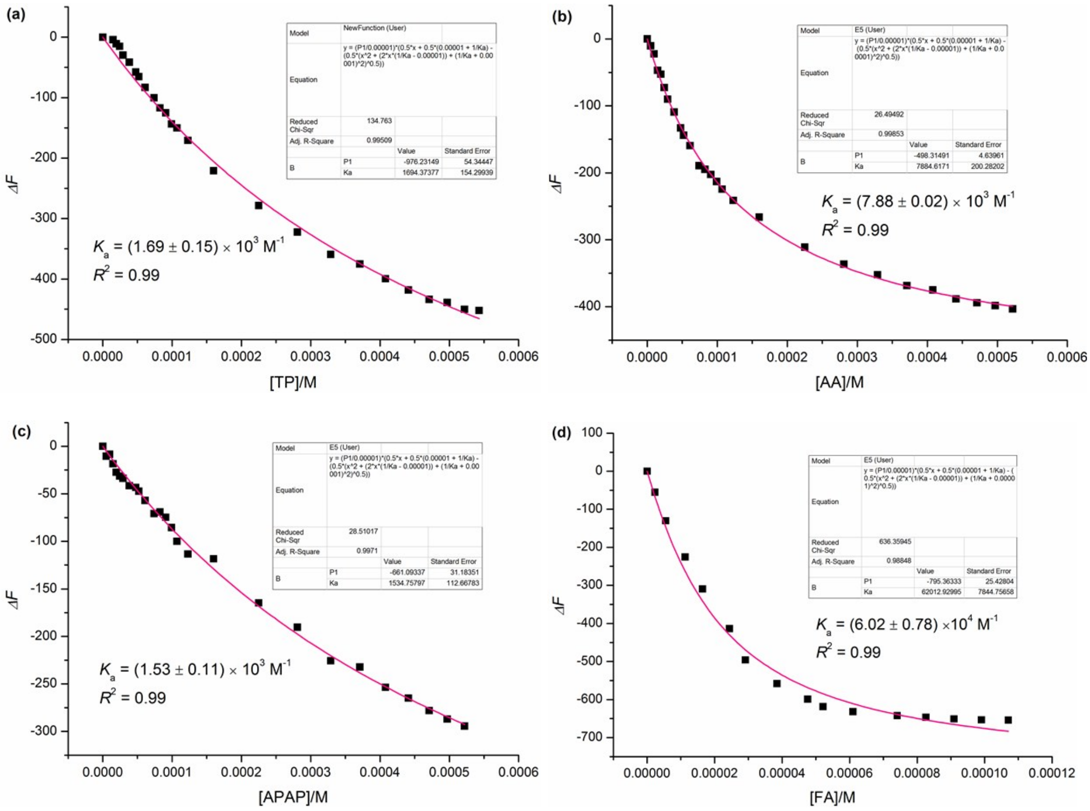

Figure S8. The fluorescence intensity changes of **CP6** upon addition of substrates (**TP, AA, APAP, FA**), respectively. The red solid line was obtained from the non-linear curve-fitting using eq. 1.

Table S1. The association constants between substrates (**TP, AA, APAP, FA**) and **CP6** by fluorescence titration experiments.

Host (guest)	K_a (L·mol^{-1})	Host (guest)	K_a (L·mol^{-1})
CP6 (TP)	$(1.69 \pm 0.15) \times 10^3$	CP6 (APAP)	$(1.53 \pm 0.11) \times 10^3$
CP6 (AA)	$(7.88 \pm 0.02) \times 10^3$	CP6 (FA)	$(6.02 \pm 0.78) \times 10^4$

References:

S1. (a) Connors, K. A. *Binding Constants*, Wiley: New York, **1987**. (b) Corbin, P. S. Ph.D. *Dissertation*, University of Illinois at Urbana-Champaign, Urbana, IL, **1999**. (c) Ashton, P. R.;

Ballardini, R.; Balzani, V.; Belohradsky, M.; Gandolfi, M. T.; Philp, D.; Prodi, L.; Raymo, F. M.; Reddington, M. V.; Spencer, N.; Stoddart, J. F.; Venturi M.; Williams, D. J. *J. Am. Chem. Soc.* **1996**, *118*, 4931−4951. (d) Zhang, J.; Huang, F.; Li, N.; Wang, H.; Gibson, H. W.; Gantzel, P.; Rheingold, A. L. *J. Org. Chem.* **2007**, *72*, 8935−8938.
